# TLR4 Expression Is Associated with Left Ventricular Dysfunction in Patients Undergoing Coronary Artery Bypass Surgery

**DOI:** 10.1371/journal.pone.0120175

**Published:** 2015-06-01

**Authors:** Orna Avlas, Arieh Bragg, Avi Fuks, James D. Nicholson, Ariel Farkash, Eyal Porat, Dan Aravot, Rachel S. Levy-Drummer, Cyrille Cohen, Asher Shainberg, Michael Arad, Edith Hochhauser

**Affiliations:** 1 The Mina & Everard Goodman Faculty of Life Sciences, Bar-Ilan University, Ramat Gan, Israel; 2 The Cardiac Research Laboratory of the Department of Cardiothoracic Surgery, Felsenstein Medical Research Center, Rabin Medical Center, Tel Aviv University, Petah Tikva, Israel; 3 Heart Failure Service and Leviev Heart Institute, Sheba Medical Center and Sackler School of Medicine, Tel Aviv University, Tel Aviv, Israel; Leiden University Medical Center, NETHERLANDS

## Abstract

**Introduction:**

Toll-like receptor 4 (TLR4) is an innate immune receptor expressed in immune cells and the heart. Activation of the immune system following myocardial ischemia causes the release of proinflammatory mediators that may negatively influence heart function.

**Aim:**

The aim of this study is to determine whether TLR4 is activated in peripheral monocytes and heart tissue taken from patients with varying degrees of myocardial dysfunction caused by coronary artery diseases and scheduled for coronary artery bypass graft (CABG) surgery before 12 months following operation.

**Methods and Results:**

Patients (n = 44) undergoing CABG surgery having left ventricular ejection fraction ≤ 45% (‘reduced EF’, n = 20) were compared to patients with preserved EF >45% (‘preserved EF’ group, n = 24). ‘Reduced EF’ patients exhibited increased TLR4 expression in monocytes (2.78±0.49 vs. 1.76±0.07 rMFI, p = 0.03). Plasma levels of C-reactive protein, microRNA miR-320a, brain natriuretic peptide (pro BNP) and NADPH oxidase (NOX4) were also significantly different between the ‘preserved EF’ and ‘reduced EF’groups. Elevated TLR4 gene expression levels in the right auricle correlated with those of EF (p<0.008), NOX4 (p<0.008) and miR320, (p<0.04). In contrast, no differences were observed in peripheral monocyte TLR2 expression. After CABG surgery, monocyte TLR4 expression decreased in all patients, reaching statistical significance in the ‘reduced EF’ group.

**Conclusion:**

TLR4 is activated in peripheral monocytes and heart tissue obtained from patients with ischemic heart disease and reduced left ventricular function. Coronary revascularization decreases TLR4 expression. We therefore propose that TLR4 plays a pathogenic role and may serve as an additional marker of ischemic myocardial dysfunction.

## Introduction

Both acute myocardial ischemia-reperfusion and chronic ischemic damage activate the immune system [1]. Molecular and cellular mechanisms underlying cardiac ischemia-reperfusion injury involve reactive oxygen species (ROS) generation, activation of endothelial cells, initiation of complement binding, increased vascular permeability, and a rapid accumulation of neutrophils [2]. Neutrophils mediate cardiomyocyte death by causing vascular plugging, releasing proteases, and ROS generation during respiratory burst [3]. Neutrophil-induced myocardial injury promotes mitochondrial permeability transition, subsequent ATP depletion, and cell death [4]. Several studies have demonstrated that the innate immune system is associated with the development of heart failure (HF)[1,5]. It has also been reported that levels of the cardio-depressant cytokines tumor necrosis factor α (TNF-α) and interleukin 6 (IL-6) are elevated in peripheral blood mononuclear cells of patients with HF [6]. These cytokines can be produced by marginating and infiltrating monocytes and may contribute to the inflammatory and subsequent immune responses undergoing HF [7,8].

Toll-like Receptors (TLRs) serve as innate immune system pattern recognition receptors, responding to inter- and intra-cellular molecules typically associated with pathogens. Among these receptors, TLR4 is activated by bacterial lipopolysaccharide (LPS) and is therefore known as the LPS receptor [7]. TLR4 is also activated by several endogenous ligands associated with tissue injury such as High-Mobility Group Box 1 Protein (HMGB1), HSP60/70, and hyaluronan in response to myocardial infarction (MI) [9]. TLR4 is expressed and elevated following ischemic injury in both leukocytes and cardiac myocytes [10]. Activated TLR4 signals through the canonical nuclear factor-κB (NFκB) pathway, increase the expression of proinflammatory cytokines such as tumor necrosis factor α (TNF-α) and interleukin 1β (IL-1β) [9]. Among TLR4 adaptor molecules, MyD88 was the first adaptor molecule discovered to be critical for TLR signaling (MyD88-dependent pathways) [11] and NADPH oxidase (NOX) in the production of proinflammatory cytokines by circulating mononuclear cells [12]. NOX4, an isoform of NOX, signals via MyD88 after ischemic injury by associating with TLR4 [13,14]. TLR4 activation is also known to upregulate NOX4 after rodent cerebral ischemia leading to the production of ROS [14]. We reported that the deficiency of TLR4 signaling reduces MI and MI-induced inflammation pathways [10]. A recent experimental study has reported that monocyte TLR4 expression is upregulated in patients with HF after acute MI [15]. TLR4 is elevated at both the mRNA and protein level in mononuclear cells of patients with coronary artery disease [16]. While the upregulation of TLR2 depends on the disease severity in patients with acute myocardial ischemia and cardiogenic shock [17], our results show that this receptor is not involved in ischemic liver damage [18].

MicroRNAs (miRNAs) are 22-nucleotide RNAs that interact with regions of mRNA for their target genes, negatively regulating target gene mRNA stability and affecting protein translation [19]. Each miRNA is estimated to influence the expression of hundreds of target genes, thereby regulating key cellular processes including survival and differentiation. The mechanism of how circulating miRNAs are released into the circulation is unclear. However, increasing evidence suggests that miRNAs are actively secreted in microvesicles [20]. Recent studies have suggested that circulating myocardial-derived miRNAs might be useful as potential biomarkers for infarction [21]. MiRNAs are present in the circulation in a stable form [22] and abnormal levels of specific miRNAs in the circulation have been reported in various conditions including HF [23]. It has recently been reported that the deregulation of miR-320a expression contributes to ischemic heart disease [19]. A correlation between the elevated serum miR-320a and the clinical prognostic parameters of systolic HF patients has also been noted [24].

We have shown that TLR4 activation during anoxic cardiac injury occurs at two separate sites of action that both contribute to cardiac dysfunction. TLR4 localized on macrophages and other infiltrating leukocytes as well as TLR4 found on cardiomyocytes causes these cells to secrete cytotoxic mediators that play a role in myocyte damage [10]. TLR4 mediates postinfarct maladaptive LV remodeling and impairs cardiac function after myocardial infarction probably via inflammatory cytokine production and matrix degradation [25].

Given the relationship between inflammatory processes and the development of left ventricular dysfunction in experimental cardiac ischemia, we designed this study to determine whether TLR4 signaling is activated in peripheral monocytes and heart tissue obtained from patients with varying degrees of myocardial dysfunction caused by obstruction of the coronary arteries before and following surgery. Expression of TLR4 and its cooperative signaling partner NOX4 were tested in auricles obtained during CABG surgery together with recognized biomarkers of inflammation and heart dysfunction such as TLR2, BNP, CRP and TNF-α. The tested tissue biomarkers together with the potential sera biomarker such as miR-320 were correlated with TLR4 expression to determine whether TLR4 may also serve as a marker for inflammation associated with cardiac dysfunction prior to and in a year following revascularization [26,27,28]. The reversibility of this process after coronary revascularization was tested by measuring EF concomitantly with TLR4, CRP and LDH.

## Materials and Methods

### Study population and design

The study was approved by the Ethical Committee of the Rabin Medical Center and written informed consent was obtained from all patients who participated in the study (0302-10-RMC). The investigation conforms with the principles outlined in the Declaration of Helsinki" (Br Med J 1964;ii:177). A total of 44 patients from the Cardiothoracic Surgery Department, Beilinson Hospital (Israel), scheduled for elective CABG surgery were enrolled in this study. Exclusion criteria included clinical signs of acute coronary syndrome, cardiogenic shock, acute infection, severe renal failure, significant valvular heart disease, or having malignancy or cardiac cachexia.

### Blood samples tests

Blood samples were obtained from each patient immediately before CABG surgery to measure serum biomarkers and to extract leukocytes to test TLR4 and TLR2 protein expression on circulating monocytes. NT-proBNP (BNP) was measured in plasma using the Roche NT-proBNP kit (Cat#04842472, Roche, Switzerland) with the Roche Cobase 411 instrument (The Cobas 4000 analyzer, Roche Diagnostics, Basel, Switzerland). C- reactive protein (CRP) and lactate dehydrogenase (LDH) were determined in the plasma using Olympus OSR6199 immuno-turbidimetric and OSR6126 Kinetic UV (Tokyo, Japan) determination tests.

### Myocardial Tissue

A section of the right atrial appendage (auricle) ~0.5 cm in size which is normally removed during open heart surgery was immediately quick-frozen in liquid nitrogen for RNA extraction and immunostaining.

### Echo-Doppler studies

Echo-Doppler studies were performed at the echocardiography unit by 3 randomly assigned technicians, using the Phillips iE33 Echo-Doppler System.

Follow-up: After surgery, two patients from the reduced EF group suffered from hemodynamic compromise requiring assist device implantation but expired during hospitalization. All other patients were invited for re-evaluation 1 year after surgery in order to investigate leukocyte TLR4 expression, serum CRP and LDH levels and to undergo an echo-Doppler study. Only 19 agreed to be studied and to donate blood.

### Monocyte isolation and flow cytometric analysis

Peripheral blood (10 mL) was obtained for the isolation of monocytes. Monocytes were purified by density gradient centrifugation over Ficoll-Paque (Lymphoprep #07801, STEMCELL Technologies, Vancouver, Canada). Monocytes were incubated with PerCP conjugated mouse anti-human CD14 antibody (clone 61D3, eBioscience, Santiago, CA, USA). The proportion of TLR4 in this separated cell fraction was examined by FACS analysis using Mouse IgG2a IsoControl (eBioscience #53-4724-80) and Anti-human CD284 (TLR4) Clone HTA125 (eBioscience #53-9917-41) or Anti-human CD282 (TLR2) Clone T2.5 (eBioscience #25-9024-80). Cells were analyzed in a FACSCalibur (Becton-Dickson, NJ, USA) and were initially gated on the basis of forward and side scatter characteristics. Cell Quest Pro Software was used to analyze results. Results are expressed as relative and mean fluorescence intensity (rMFI).

### mRNA quantification by Real-Time PCR

Total RNA was extracted from heart auricles by the TRI Reagent solution (Ambion AM9738, Carlsbad, CA, USA). RNA was diluted to a 100 ng/μl mixture and converted to cDNA using the Applied Biosystems High Capacity Reverse Transciption Kit (Part #4368814) (Applied Biosystem Inc., Foster City, California, USA). We analyzed TLR4, BNP, and TLR2 mRNA expression levels using the quantitative real-time RT-PCR method as previously described [29].

RT-PCR using primer pairs for TLR2, TNF-α, BNP, TLR4, and NOX4 were purchased from Applied Biosystems (Cat. #s Hs00152932_m1; Hs01113624_g1; Hs00173590_m1; Hs01060206_m1; Hs00418356_m1, respectively). The thermal cycling conditions included an initial step at the holding phase of 20’ at 95°C followed by 40 cycles of a cycling stage of 1’ at 95°C and 20’ at 60°C. Gene expression analysis was performed using the Applied Biosystems Sequence Detection program using the ΔΔCT method. The samples tested were normalized to the housekeeping gene glyceraldehyde 3-phosphate dehydrogenase (GAPDH) (Applied Biosystems, Cat #Hs99999905_m1).

### Immunofluorescence staining and imaging

Frozen human auricle specimens were stored at -80°C until use. The specimens were fixed 24 hours in ice cold 4% paraformaldehyde pH 7.4, immersed overnight in 30% sucrose in PBS, then cryosectioned at 5 μm thickness. All immunostaining used 0.2% triton X-100 (Sigma) in PBS. The sections were transferred directly onto slides, treated 15 minutes with 1% sodium borohydride in water to reduce autofluorescence, washed 1h in PBS and blocked for 1h with 2% normal donkey serum in PBS. Slides were then incubated sequentially with antibodies as follows: overnight at 4°C 1:500 goat anti-human TLR4 IgG (Cat #AF1478, R&D Systems, Minneapolis, MN, USA) followed by 1h 1:1000 donkey anti-goat IgG NL557-conjugated antibody (Cat NL001, R&D Systems, Minneapolis), overnight 4°C 1:1000 mouse anti-cardiac troponin I antibody (Cat #ab47003, Abcam, Cambridge, MA), 1h 1:2000 donkey anti-mouse IgG (Cat #715-166-150, Jackson Immunoresearch Laboratories, West Grove, PA). After immunostaining, the slides were washed briefly with PBS, stained 15 minutes with 4,6-diamidino-2-phenylindole (DAPI) (H-1200 Vector labs Inc., Burlingame, CA, USA) then mounted using Dako anti-fade mounting media (Cat #S3023, Dako Denmark A/S, Glostrop, Denmark).

The slides were viewed and photographed using an Olympus BX52 fluorescent microscope and DP71 camera (Olympus Microscopes, Center Valley, PA. USA). Images were processed using ImageJ (http://imagej.nih.gov/ij/, National Institutes of Health, USA).

### miRNA extraction and RT-PCR

MicroRNAs were extracted from plasma using QIAzol lysis reagent and the miRNeasy Serum/Plasma kit (Cat #217184, Qiagen Inc., Alameda, CA, USA) and converted to cDNA using the miScript PCR Starter kit following the manufacturer's instructions (Cat #218193, Qiagen Inc., Alameda, USA). RT-PCR was performed on an StepOnePlus RT-PCR machine (Applied Biosystems, Foster City, California, USA) to detect changes of miR320a and miR15a using commercially available primers following the manufacturer's instructions (Cat #hsa-miR-320a; 5'AAAAGCUGGGUUGAGAGGGCGA, Cat# Hsa_miR-15a; 5'UAGCAGCACAUAAUGGUUUGUG, Qiagen Inc., Alameda, CA, USA). miR320a and Mir15a were normalized the small nuclear RNA U6 (snU6) 5'- CGCAAGGATGACACGCAAATTC. A 9 μl volume (comprised of a mixture of SYBR Green Master Mix, Universal Primer, Primer Assay, and RNase-free water) was added to a 1 μl volume of cDNA (following the structure of miRNeasy Serum/Plasma Handbook, Qiagen). The platter-PCR protocol consisted of the following steps: a single hold phase of 15 minutes at 95°C followed by 40 cycles each of the following:15’ at 94°C, 30’ at 55°C, and 30’ at 70°C. Data was collected at the end of each cycle. Quantitation was by the ΔΔCT method.

### Data analysis

The study population was split into 2 categories: patients with preserved left ventricular ejection fraction (EF>45%) and patients with reduced left ventricular ejection fraction (EF≤45%). Statistical analyses were performed using the SPSS version 20 statistical software (SPSS Inc., Chicago, IL). All continuous values are presented as mean±SD. The Kolmogorov-Smirnov test for normal distribution was performed for all continuous variables. Unpaired and paired t test and Pearson correlations were performed, as appropriate for normally distributed data. Spearman's correlation coefficients had to be used to examine the relationship between TLR4 levels, cytokine concentrations and EF. The Fisher Exact test was used to study associations between categorical variables. A 2-tailed value of P <0.05 was accepted as statistically significant.

## Results

### Baseline characteristics

Patients in the ‘reduced EF’ group had mean EF 37±7% (n = 20) compared to 57±4% in the ‘preserved EF’ group (n = 24, P = 0.0001). There were no significant differences between the two groups in mean age, gender, white blood cell (WBC) count, differential count or serum electrolytes (see Table 1A). Patients were treated with statins, anti-platelet agents, beta blockers, ACE inhibitors and other medications without significant differences between the groups (Table 1B).

**Table 1:** Baseline characteristics of the study population (A) and medication (B).

*A*. *Clinical characteristics*	‘reduced EF’, n = 20	‘preserved EF’ n = 24	P value
**Age**	58±9	65±9.2	NS
**Female (%)**	5	9	NS
**Ejection Fraction (%)**	36.6±6.9	56.8±4.0	0.0001
**Monocytes (%)**	4.6±2.9	4.9±2.9	NS
**Smoking (%)**	27	28	NS
**Diabetes (%)**	40	38	NS
**State post myocardial infarction (%)**	60	60	NS
**Serum Creatinine (mg/dl)**	0.9±0.2	1.0±0.4	NS
**Serum Sodium mmol/L**	137±1.8	138±2.0	NS
**White blood cells count (K/micl)**	9.3±1.4	8.4±2.2	NS
***B*. *Medication***			
**Beta-blockers**	14/20 (70%)	18/24 (75%)	NS
**Ace-inhibitor/ ARB**	13/20 (65%)	17/24 (70.8%)	NS
**Calcium blockers**	1/20 (5%)	7/24 (29.2%)	NS
**Statins**	15/20 (75%)	20/24 (83.3%)	NS
**Anti-platelet**	15/20 (75%)	20/24 (83.3%)	NS
**Anti-arrhythmic**	1/20 (5%)	0/24 (0%)	NS
**Diuretics**	5/20 (25%)	5/24 (20.8%)	NS
**Nitrates**	3/20 (15%)	3/24 (12.5%)	NS
**Diabetics oral**	5/20 (25%)	5/24 (20.8%)	NS
**Diabetics insulin**	3/20 (15%)	4/24 (16.7%)	NS
**Anti-coagulation**	2/20 (10%)	4/24 (16.7%)	NS
**Steroids**	1/20 (5%)	0/24 (0%)	NS

### Biomarkers in patient blood

**Fig 1:**
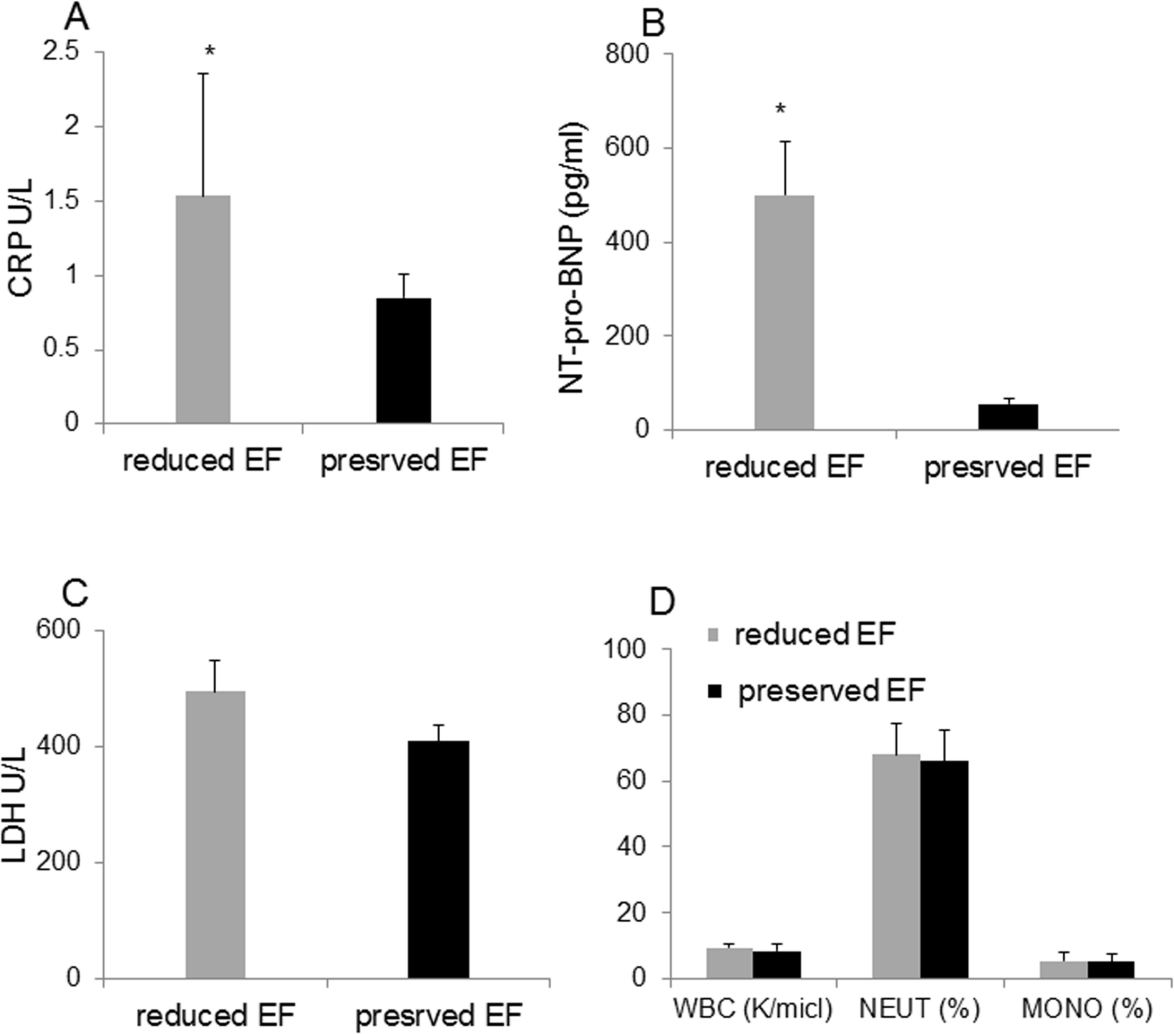
Biomarkers in the plasma and white blood cells count. Protein expression of different stress biomarkers in patients before CABG surgery: ‘reduced EF’ and ‘preserved EF’ patients. (A) CRP, p = 0.038, (B) NT Pro-BNP, *P<0.0001, (C) LDH, p = 0.497 reduced versus ‘preserved EF’, (D) WBC/Monocyte/Neutrophils count of the 2 groups, no statistical difference was observed.

The plasma concentration of NT-proBNP and CRP were significantly elevated in ‘reduced EF’ patients compared with ‘preserved EF’, P<0.05 (Fig 1A and 1B). All patients with EF≤45% had NT-proBNP above 75pg/ml, while patients with EF above 45% had NT-proBNP values in the normal range, i.e. below 75pg/ml. No significant difference was found in LDH levels between the two groups (Fig 1C). Plasma NT-proBNP correlated inversely with the EF (r = -0.638, P<0.0001) and with CRP (r = -0.394, P<0.013). Fig 1D shows the WBC counts and the percentage of monocytes and neutrophils in the study groups (Fig 1D).

### Expression of TLR4 and TLR2 on patient monocytes

**Fig 2:**
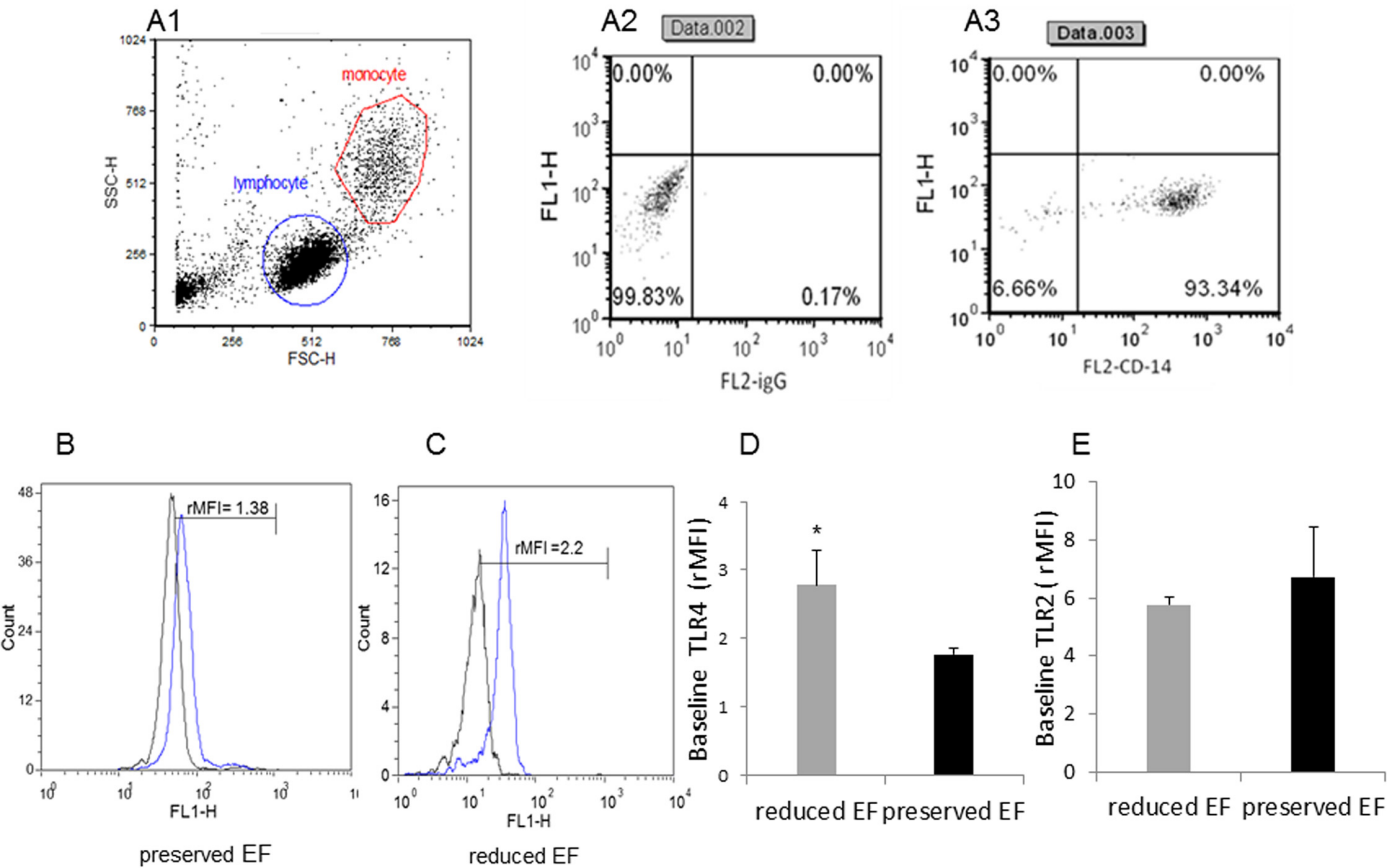
Dot plot and histograms representing TLR4 and TLR2 levels in monocytes. (A1-A3) Monocytes/lymphocyte cell population after CD14-positive cell staining. (B-C) Shift to the right demonstrating an increase in the amount of TLR4 staining in the monocytes. Specific mean fluorescence (MFI) can be quantified from these histograms for each case. (D) TLR4 levels on the population of monocytes as measured using relative mean fluorescence intensity (rMFI). (C) ‘reduced EF‘ patients (EF<45%) and ‘preserved EF‘ patients (EF>55%). (E) TLR2 levels on the population of monocytes as measured using rMFI. FACS analysis,* P<0.03 patient with ‘reduced EF’ TLR4 was higher than that of patients with ‘preserved EF’.

FACS analysis for CD14-positive circulating monocytes tested if TLR4 and TLR2 expression was elevated in our patient populations (Fig 2). Mean TLR4 expression was higher in ‘reduced EF’ patients compared to ‘preserved EF’ patients (P<0.03). Whereas a borderline correlation was found between TLR4 expression in monocytes and the EF (P = 0.052), a significant correlation between TLR4 and NT-proBNP (P<0.006), (Fig 2A–2D) was noted. There was no difference in TLR2 expression between the two groups, (see Fig 2E).

### Expression of miR320a in patients’ serum

**Fig 3:**
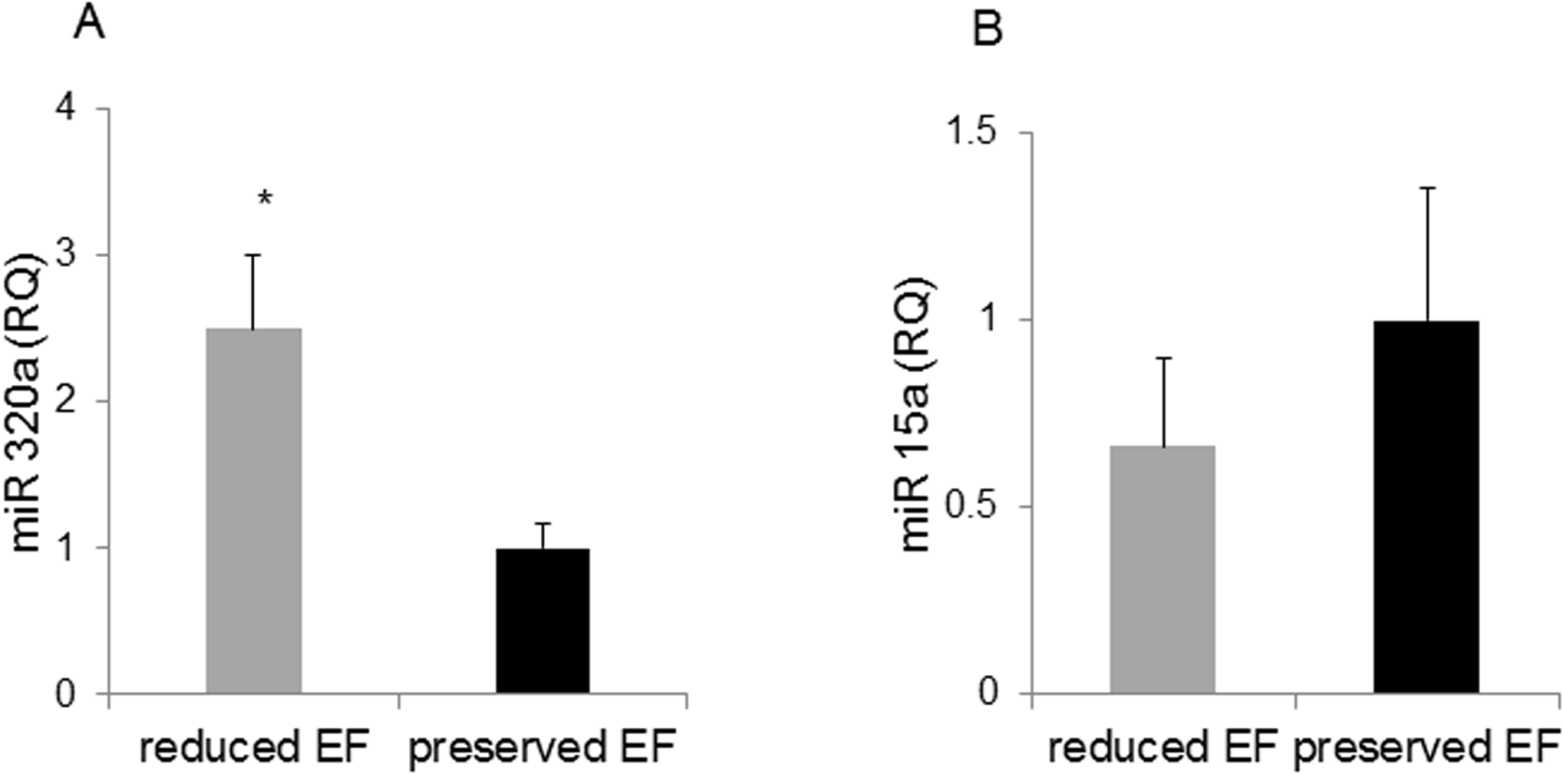
miR expression. A. miR320a expression in the serum of patients undergoing CABG surgery, with reduced EF was higher than preserved EF, * P <0.001. B. The miR15a, is presented in Fig B and noted as unchanged.

Since elevated serum miR-320a was reported as a clinical prognostic parameter of systolic HF patients we correlated its level with the two groups of patients. Patients with ‘reduced EF’ exhibited a 2.5-fold elevated miR-320a compared to the ‘preserved EF’ patients (Fig 3A, P = 0.001). There was a significant inverse correlation between the miR-320a and the EF values (r = -0.736, P<0.003). There was also a significant positive correlation between miR-320a and TNFα (r = 0.711, P<0.01) and miR-320a and TLR4 (r = 0.471, P<0.04). No difference between groups was found for the expression of the microRNA miR-15a. (Fig 3, P>0.05).

### Myocardial expression of TLR4 signaling in ‘preserved EF’ vs. ‘reduced EF’ patients

**Fig 4:**
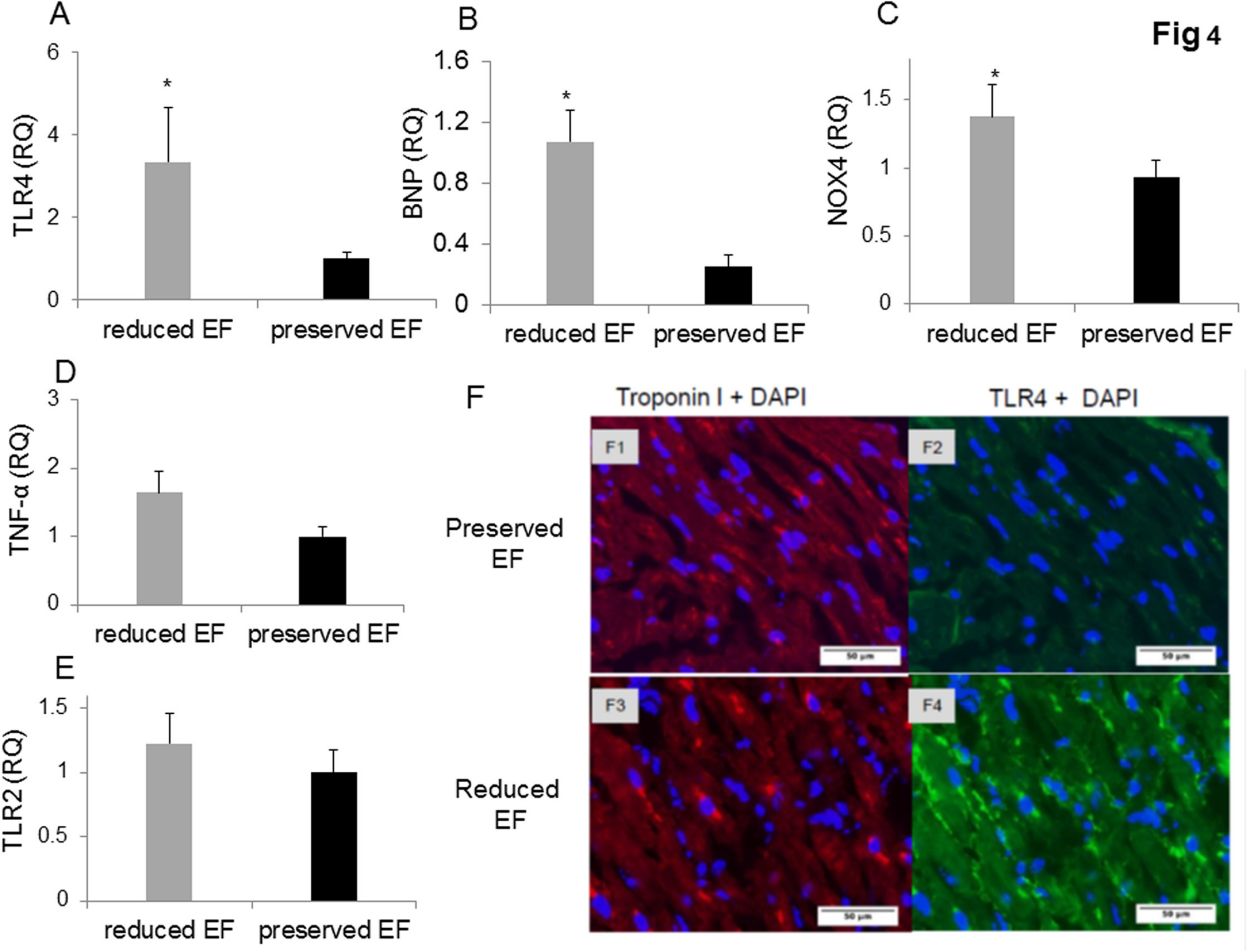
Gene expression of biomarkers of injury and immunostaining of TLR4 in patient auricles. (A-D) TLR4 and NOX4 are activated resulting in elevated TNF-α in the auricles. Auricles obtained during CABG surgery presented higher expression of TLR4 (P<0.03), BNP (P<0.05), NOX4 (P<0.03), and TNF-α (P = 0.135) in reduced versus ‘preserved EF’. (E) TLR2 expression was similar in both groups. (F1-F2) Representative photographs show double-immunostaining of Troponin I and TLR4 in ‘preserved EF’ auricle. (F3-F4) Representative photographs show double-immunostaining of troponin I and TLR4 in the ‘reduced EF’ auricle. TLR4 staining revealed an apparent upregulation in all ‘reduced EF’ patients examined compared to ‘preserved EF’ patients' tissue.

RT-PCR for TLR4 mRNA in the atrial appendage tissue revealed a 3-fold upregulation in the ‘reduced EF’ patients compared to the ‘preserved EF’ patients (Fig 4A) (P<0.03). BNP mRNA was markedly elevated in patients with reduced EF (Fig 4B) (P< 0.005). NOX4 was also elevated in ‘reduced EF’ patients (P< 0.03) (Fig 4C). TNFα mRNA was elevated but did not reach statistical significance, P = 0.13, (Fig 4D). However, TLR4 and TNFα significantly correlated with each other (r = 0.689, P<0.001). There was no significant difference in TLR2 gene expression between the preserved and reduced EF groups (Fig 4E).

There was a significant inverse correlation between TLR4 and EF(r = -0.5, P<0.008) and NOX4 and EF (r = -0.356, P<0.05).

There was a significant correlation between NOX4 and TLR4 (r = 0912, P<0.0001) and NOX4 and TNFα (r = 0.732, P<0.001), indicating that TLR4, NOX4, MIR320 and TNFα are all related to each other in the cardiac tissue with low EF.

We examined TLR4 expression in patients' auricular appendage by double-immunostaining for TLR4 and troponin I. We examined 6 heart sections from each patient group and surveyed the entire section microscopically. TLR4 staining revealed an apparent upregulation in all ‘reduced EF’ patients examined compared to ‘preserved EF’ patients' tissue. TLR4 was expressed on the surface of cardiomyocytes as determined by troponin I staining (see Fig 4, F1-F4) for representative images.

### Post-surgical TLR4 expression

Two patients with ‘reduced EF’ developed post-surgical HFand died during hospitalization, had a relatively high TLR4 monocyte expression (2.35 and 2.45 rMFI compared to 1.76±0.07 rMFI in the 'preserved EF'). Follow-up blood samples were available for analysis in 19 patients (9 from ‘reduced EF’ and 10 from ‘preserved EF’). Left ventricular revascularization led to a slight, non-significant improvement of left ventricular EF in the ‘reduced EF’ group (improved from 34% to 38%). Monocyte TLR4 expression decreased after surgery in both groups (Table 2) reaching statistical significance in the ‘reduced EF’ group. There were no statistical differences in CRP and LDH after surgery between the reduced and preserved EF groups (Table 2).

**Table 2:** Comparison of a 12-month post-surgical follow up to the baseline data.

	‘reduced EF’ n = 11		‘preserved EF’ n = 9	
	Baseline	Follow-up	Baseline	Follow-up
**EF (%)**	34.85±7.08	38.18±10.3	57.76±3.59	60±1
**TLR4 (MFI)**	2.12±0.05	1.83±0.22*	1.63±0.13	1.45±0.60
**CRP (mg/dL)**	1.78±1.29	0.65±0.74	0.44±0.48	0.36±0.31
**LDH(U/L)**	526±216	381±69	387±134	369±39.6

Legend for Table 2:

*P = 0.03 vs baseline

## Discussion

This work evaluated the contribution of immune and myocardial TLR4 expression on acute cardiac depression in cardiac patients suffering from left ventricular dysfunction. We found that TLR4 together with plasma BNP were significantly elevated in patients with ischemic left ventricular dysfunction. The results of this study suggest that activated TLR4 signaling may play an important role in the progression of the failing heart as evidenced by: (1) elevated TLR4 levels in monocytes and myocardial tissue in patients with ‘reduced EF’ relative to those with ‘preserved EF’; (2) no upregulation of TLR2 was found in both monocytes and tissue. TLR4 continuing activation leads to the secretion of proinflammatory cytokines and activation of cardiac stress biomarkers as measured by gene or protein levels of NOX4 and miR-320 and proBNP. TLR4 and TNFα significantly correlated with each other (P<0.001). (3) Importantly, our observations post CABG suggest reversibility of this process after coronary revascularization. TLR4, CRP and LDH were reduced probably due to the beneficial effect of the revascularization procedure.

Our analysis supports previous reports that the inflammatory response following myocardial ischemia is an independent predictor of left ventricular dysfunction, specifically in subjects with ischemic heart disease [30]. Peripheral monocytosis is associated with LV dysfunction after acute MI, aortic valve inflammation process, stenosis, and atherosclerosis [31]. Asp299Gly polymorphism in the TLR4 gene, which impairs inflammatory responses, is associated with reduced vascular inflammation, assessed by C-reactive protein and a decreased risk for coronary artery disease and diabetes [32]. We reported that the heart and lymphocytes express TLR4 following LPS or ischemic insult and secrete TNF-α and IL-1β in mice [10,18,29]. In the current work, we observed an inverse relationship between TLR4 expression and ejection fraction, in both monocytes and cardiac tissue. It appears that TLR4 is particularly important for continuous monocyte activation in heart dysfunction, and that TLR4 overexpression is accompanied by secretion of proinflammatory cytokines such as CRP and TNF-α, as well as a known biomarker of cardiac failure—pro BNP. Although there was a positive association of TLR4, there was a much stronger correlation of proBNP with reduced cardiac performance.

TLR4 mRNA of reduced EF patients was upregulated 3.4-fold in heart tissue compared to activation by only a 23% increase in monocytes. While the former reflects cardiac gene expression, the latter correlates with expression of the protein in leukocytes. Immunohistochemical TLR4 staining in cardiac tissue was more intense for ‘reduced EF’ patients than for ‘preserved EF’ patients, demonstrating that TLR4 correlation with myocardial dysfunction gene and protein expression does not always rise proportionally. The main pathway through which TLR4 signaling induces cardiac inflammation ultimately leads to NF-κB activation in immune cells and cardiomyocytes leading to cytokine secretion and elevated inflammatory markers. Although TNFα was not statistically different between preserved and reduced LV function there was a significant correlation between TLR4 and TNFα (P<0.001). TNFα was statistically higher in wild type hearts compared to knockout TLR4 hearts subjected to ischemia [10]. Collectively, our data demonstrates that due to ischemic cardiac injury there is both cardiac and systemic TLR4 activation contributing to the reduced contractility of the heart as seen by the reduced EF. Despite the fact that the right auricle is remote from the left ventricle, the mechanism for this upregulation remains to be determined. This TLR4 augmentation could be triggered by atrial ischemia or by elevated filling pressures as a result of ventricular ischemia.

Since there was no difference in the white blood cell count between the two groups of patients, our findings in peripheral monocytes reflects the fact that upregulation of TLR4 in ‘reduced EF’ patients results from increased TLR4 expression on individual cells. It should be noted that monocyte TLR4, but not TLR2, is upregulated in ischemic heart disease patients with ‘reduced EF’. Overexpression of TLR4 in the immune cells is involved in the development of left ventricular dysfunction [30]. Although it is documented that TLR2 participates in cardiac damage following ischemia [17], our present results show that this receptor is not involved in the prognosis of heart dysfunction in coronary arteries disease patients. We performed FACS analysis on the blood samples together with RTPCR on the heart samples and both measurements resulted in no differences. Using a model of liver ischemia reperfusion we also found that TLR2 was not involved (18).

We examined the relationship of monocyte TLR4 expression with improved heart function one year following surgery. While the average EF was not significantly affected by surgery, reduction of the ischemic burden was associated with a significant decrease in the amount of TLR4 expressed by the monocytes (Table 2). Following revascularization, TLR4 activation was significantly reduced, meaning that increased TLR4 expression is reversible and correlated with the degree of cardiac ischemia. In patients with ischemic cardiomyopathy the improvement in LVEF after CABG is often incomplete and takes time, if it occurs at all. The infarct area which is irreversible leads to loss of function that is permanent in many cases. Therefore, TLR4 can be reduced with no improvement in EF. Regretfully, not all patients came for the follow-up and consequently only a small number of patients were available for comparison. Perhaps a longer follow-up period would provide more information [33].

NOX4 has been shown to be upregulated and co-localized with TLR4, after cerebral ischemia and reperfusion in mice and in brain tissue of human stroke patients. In addition, NOX4 was co-immunoprecipitated with TLR4 after cerebral ischemia in mice [34]. Inhibiting TLR4 signaling, either genetically or pharmacologically with TAK-242 (Resatorvid), was previously shown to suppress NOX4 induction and to reduce oxidative stress [34]. In our work, NOX4 together with TLR4 were highly expressed in the myocardium of patients with ‘reduced EF’. Both NOX4 and TLR4 expression correlated with TNFα secretion resulting in reduced cardiac performance. These findings indicate that inhibiting TLR4-NOX4 signaling may be a promising candidate to treat HF.

The involvement of differentially expressed miRNAs in the inflammation that occurs during the development of HF is still subject of investigation [35].Transgenic mice with cardiac-specific overexpression of miR-320a had a larger MI size than wild-type mice after ischemia-reperfusion and inhibiting miR-320a with antagomir-320 significantly reduced infarct size [19]. In the current study, we found that high levels of miR-320a correlated with the upregulation of TLR4 expression, TNFα and reduced ejection fraction. Our results are consistent with the notion that increased circulating levels of miR-320a in HF patients may serve as a marker of myocardial ischemia [23]. As for miR15a it was found that miR-15a is slightly upregulated (about 1.5 folds) in the infarcted region of porcine cardiac tissue 24 hours after ischemic injury in the porcine MI model compared but not in the border infarct zone [36]. In our work, sera miR 15a was similar in patients with preserved and reduced LV function undergoing CABG surgery. We propose that both TLR4 and miR-320a are activated in patients with myocardial dysfunction.

Human studies have demonstrated the increased TLR4 expression in hearts of advanced HF patients [37]. The main pathway through which TLR signaling induces cardiac inflammation ultimately leads to NF-κB activation in immune cells and cardiomyocytes. NF-κB plays a critical role in the expression of genes involved in cell death, inflammatory responses and cell survival [38]. Here, we noted that inflammatory markers as well as intracellular signaling, such as NOX4 and MIR320, were differently expressed in the groups with preserved vs. reduced ejection fraction. Importantly, our observations post-CABG suggest reversibility of this process after coronary revascularization in relation to TLR4 activation. Secretion of proinflammatory cytokines as well as a known biomarker of cardiac failure such as pro BNP emphasizes the role of continuous TLR4 activation in the pathogenesis of cardiac dysfunction. Cellular Nox4 upregulation reinforces our finding that continuous intracellular signaling of TLR4 activation leads to impaired cardiac function. The present study identifies elevated TLR4 on peripheral monocytes as a possible marker for cardiac dysfunction caused by the obstruction of coronary arteries. We suggest that TLR4 inhibition by antisense strategies or pharmacological approaches may emerge as an alternative method in the treatment of coronary stenosis to prevent the development of contractile dysfunction and HF.

## Study Limitations

Several limitations of this work must be taken into account. The present study is a single-center small group study, so further investigations with more patients are needed to estimate the impact of TLR4 up regulation on the ischemic myocardium and HF. Although our department conducts about 600 CABG surgeries per year, the exclusion criteria of this research permitted the recruitment of only a small group of patients comprising about 10% of possible candidates during the study period. Approximately 50% of recruited patients (n = 44) refused a follow-up visit, which impeded a detailed assessment of various biomarkers along with clinical outcome data. The examination of additional groups of patients, such as acute coronary syndrome and overt HF, are required to further clarify the role of TLR4 in being the cause of or a marker for those conditions.
